# Supplementary material for: Cloud BioLinux: pre-configured and on-demand bioinformatics computing for the genomics community
Source: BMC Bioinformatics. 2012 Mar 19;13:42. doi: 10.1186/1471-2105-13-42 (PMC3372431; doi:10.1186/1471-2105-13-42)
Supplement: Additional file 1 — Supplementary 1 Cloud BioLinux software documentation in the form of a mini, self-contained website. Users need to download and uncompress the .zip file, and open through a web browser the "index.html" file available on the main directory. (ZIP 1823 kb). [file 1471-2105-13-42-S1.ZIP › Cloud-BioLinux-Package-Documentation/docs/mspcrunch.html]

Bio-Linux Software Documentation Pages

Back to search form

## mspcrunch

|  |  |
| --- | --- |
| Name | mspcrunch |
| Description | **MSPCrunch** is a Blast Post Processing file  Related resources from the Sonhammer group see: http://sonnhammer.sbc.su.se/databases.html  **References:**  Sonnhammer, ELL and Durbin, R (1994). A workbench for Large Scale Sequence Homology Analysis. Comput. Applic. Biosci, 10:301-307. [ENTREZ]    Sonnhammer, ELL and Durbin, R (1994). An expert system for processing sequence homology data. ISMB 2:363-368 [ENTREZ] |
| Homepage | http://sonnhammer.sbc.su.se/MSPcrunch.html |
| Remote Documentation | ftp://ftp.cgb.ki.se/pub/prog/MSPcrunch+Blixem/MSPcrunch2.ps.Z |
